# Development, outcome and costs of a simulation-based neurosurgery bootcamp at the national level

**DOI:** 10.1186/s12909-022-03965-9

**Published:** 2022-12-28

**Authors:** Saqib Kamran Bakhshi, Rida Ahmad, Asma Altaf Hussain Merchant, Ali Aahil Noorali, Komal Abdul Rahim, Namra Qadeer Shaikh, Noreen Afzal, Maryam Pyar Ali Lakhdir, Muhammad Shahzad Shamim, Adil Hussain Haider

**Affiliations:** 1 Medical College, Aga Khan University, Karachi City, Sindh, Pakistan; 2 Community Health Sciences, Aga Khan University, Karachi City, Sindh, Pakistan

**Keywords:** Neurosurgery, Residency, Simulation-based training

## Abstract

**Introduction:**

With a growing interest in simulation-based training to develop clinical proficiency, bootcamps have been utilized for imparting basic skills to medical trainees. While considerable research on the topic exists in high income countries, no such neurosurgical teaching standards have been employed in Low- and Middle-Income Countries.

**Methods:**

We conducted a cross-sectional study to explore the effectiveness of first low-cost, multi-center regional neurosurgery bootcamp in South Asia. Twenty-two participants attended the bootcamp and practiced 12 hands-on skills over the course of 2 days. Burr-holes and craniotomies were done on 3D printed skulls. Lumbar drain insertion was practiced on a purpose-built lumbar puncture mannequin. For laminectomy, we used an in-house designed simulation. The modified Objective Structured Assessment of Technical Skills tool was utilized for skills Assessment. Feedback from faculty and residents was collected via a standard 5-point Likert scale.

**Results:**

Only one participant (4.55%) had previously attended a neurosurgical skills workshop. Comparison of outcomes on 1^st^ and 3^rd^ attempts of cranial and spinal skills showed a significant improvement in all 14 domains assessed (*p* <0.05). Positive feedback was received ranging from 3.9 up to 4.8 on a 5-point Likert scale. Overall cost per participant culminated to $145, significantly lower than previously reported data.

**Conclusion:**

Our findings report the effectiveness of sustainable, low-cost training models which can be easily reproduced elsewhere. These indigenously designed simulators can be modified for variable difficulty level and serve as an effective educational strategy in improving learners’ skills, knowledge and confidence.

**Supplementary Information:**

The online version contains supplementary material available at 10.1186/s12909-022-03965-9.

## Background

The transition from medical school to residency training can be very challenging [1]. The delicate and complex details of neurosurgery make the switch to specialty training even more exigent. During the last few decades, neurosurgical patient care and management has witnessed significant advancements along with the increasing use of high-tech innovations [2]. This effect has trickled into training methodologies as well, to help residents develop excellence in their dexterity, critical thinking and technical skills [3]. The technological evolution in neurosurgery, coupled with the introduction of reduced working hours by the Accreditation Council for Graduate Medical Education (ACGME), has inculcated an interest in simulation-based training for residents to develop neurosurgical acumen and proficiency [3, 4].

Integrating post-graduate year-1 (PGY-1) residents into the program is often challenging for the faculty. Limited exposure to neurosurgery in medical school results in high burden of responsibility falling on program directors in devising effective strategies for building basic knowledge and skills framework of new residents. Bootcamps are a means to provide a strong footing for effective learning of residents in conducive environments [5]. The traditional master-apprentice relationship to teach specialized surgical skills to residents in the operating room, has largely been substituted and strengthened by laboratory-based cadaveric dissections and simulation-centric education. The latter has shown to be superior in enhancing one’s clinical knowledge and skills without compromising patient safety, and can be reproduced elsewhere [6]. While bootcamps have long been utilized for imparting basic skills to the graduating medical students and interns, they have only recently been employed for specialties like neurosurgery [7–9].

Having numerous advantages, lab training and bootcamps have become a regular feature for new PGY-1 residents in nearly all high-income countries, however, its implementation in the low- and middle-income countries (LMICs) is still limited. Disparity in the standards of training at different centers within the same country make it difficult to devise a bootcamp curriculum that covers the requirements of all facilities. Moreover, high cost associated with its implementation often puts developing and underdeveloped countries at a disadvantage in adopting them as part of their surgical curriculum [8, 10]. Only 6% of neurosurgical trainees have access to simulation laboratories in Southeast Asia, while the rest are deprived of this learning resource [11]. Limited resources and low gross domestic product (GDP) per capita in LMICs make high cost simulated training unsustainable. Employing cadaveric dissection in bootcamps and surgical training labs could have been a low-cost alternate, but in most LMICs the absence of regulatory laws and religio-cultural norms significantly limit access to cadavers.

Standardization and improving quality of neurosurgical care in LMICs warrants establishment of a low-cost training model for residents, that could be easily replicated on a large scale. Since no such neurosurgical teaching standards have been earlier employed in this region, there is a dearth of data on the effectiveness of bootcamps incorporating simulation-based methods in significantly resource limited settings. To overcome this disparity, we conducted the first low-cost, multi-center regional neurosurgery bootcamp in South Asia. It was dedicated to the upskilling of PGY-1 neurosurgery residents, and aimed to establish a sustainable neurosurgical skills’ curriculum for developing countries. The objective of this study was to assess the effectiveness of this structured bootcamp on the knowledge and procedural skills of the participating neurosurgery residents.

## Methods

This was a cross-sectional study conducted as part of the first regional neurosurgery bootcamp organized at the Aga Khan University Hospital (AKUH), Pakistan. The bootcamp was held over two days. Twenty-two PGY-1 residents from five neurosurgery training centers attended the bootcamp. These five centers are registered with the College of Physicians and Surgeons Pakistan (CPSP), which is the educational body regulating all postgraduate training programs in the country. Approximately 25% of neurosurgical residents within the country are represented by these institutions, with each having its individual structure of training. To standardize and congregate this diversity, skills training at the boot camp was conducted by senior faculty hailing from all the participating institutes. This study was approved by the Ethical Review Committee [ERC] of the Aga Khan University Hospital, Pakistan (reference # 2022-7251-20815). A written informed consent was taken from every participant of the study. All the methods were performed in accordance with relevant guidelines and regulations. All participants who provided written informed consent and attended the full duration of the bootcamp were included in the study.

### Bootcamp curriculum

The bootcamp curriculum comprised of a workshop with hands-on skill stations. It was modelled on the SNS neurosurgery bootcamp curriculum, which has been approved by ACGME, and is being used in the mandatory bootcamps for neurosurgery PGY-1 residents in the US [5, 12]. All participants attended the workshop and practiced 12 hands-on skills as shown in Table 1. There were 7 stations of hands-on skills with a supervising faculty present at each station. All skills were first demonstrated by the faculty, followed by participants performing them at least thrice. Burr-holes and craniotomies were done on 3D printed skulls made with Acrylonitrile Butadiene Styrene (ABS) and having a melting point of 145.2 degrees Fahrenheit. Lumbar drain insertion was practiced on a purpose-built lumbar puncture mannequin. For laminectomy, an in-house designed simulator with the provision of incorporating depth perception was used. Saw-bone spine model was used for laminectomy with bone nibbler and Kerrison ronguer.

**Table 1:** Bootcamp curriculum for hands-on skills

**Hands-on Skills**	
External Ventricular Drain insertion	Handling of the operative microscope
Intracranial pressure monitoring	Microdissection of cow brain
Ventriculo-peritoneal shunt insertion	Simulated lumbar drain insertion
Neuro-navigation for cranial cases	Simulated lumbar aminectomy
Simulated burr-holes on 3D skulls	Simulated spine dural closure
Simulated craniotomy on 3D skulls	Basic Neurosurgical instrumentation

### Skills’ assessment

**Fig 1:**
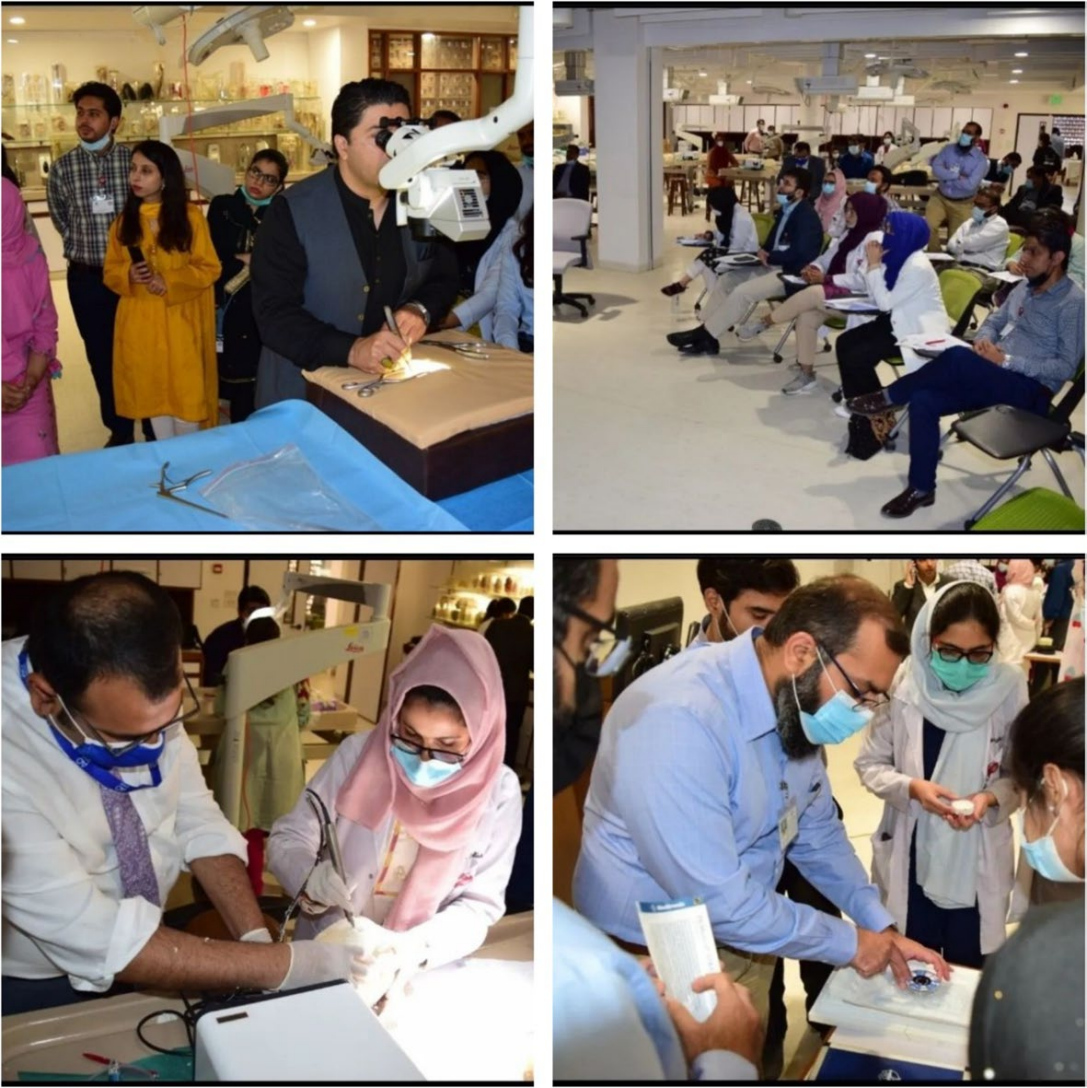
Faculty teaching residents at the bootcamp

Each resident was assigned a unique identification number which was used for data collection to maintain confidentiality. Evaluation of procedural and surgical skills was done for each participant using the modified Objective Structured Assessment of Technical Skills (mOSATS) tool [13]. The four most important skills were evaluated by the faculty (burr-holes, craniotomy, lumbar drain insertion and laminectomy). Figure 1 includes some pictures from the bootcamp showing residents being taught by the faculty.

**Fig 2:**
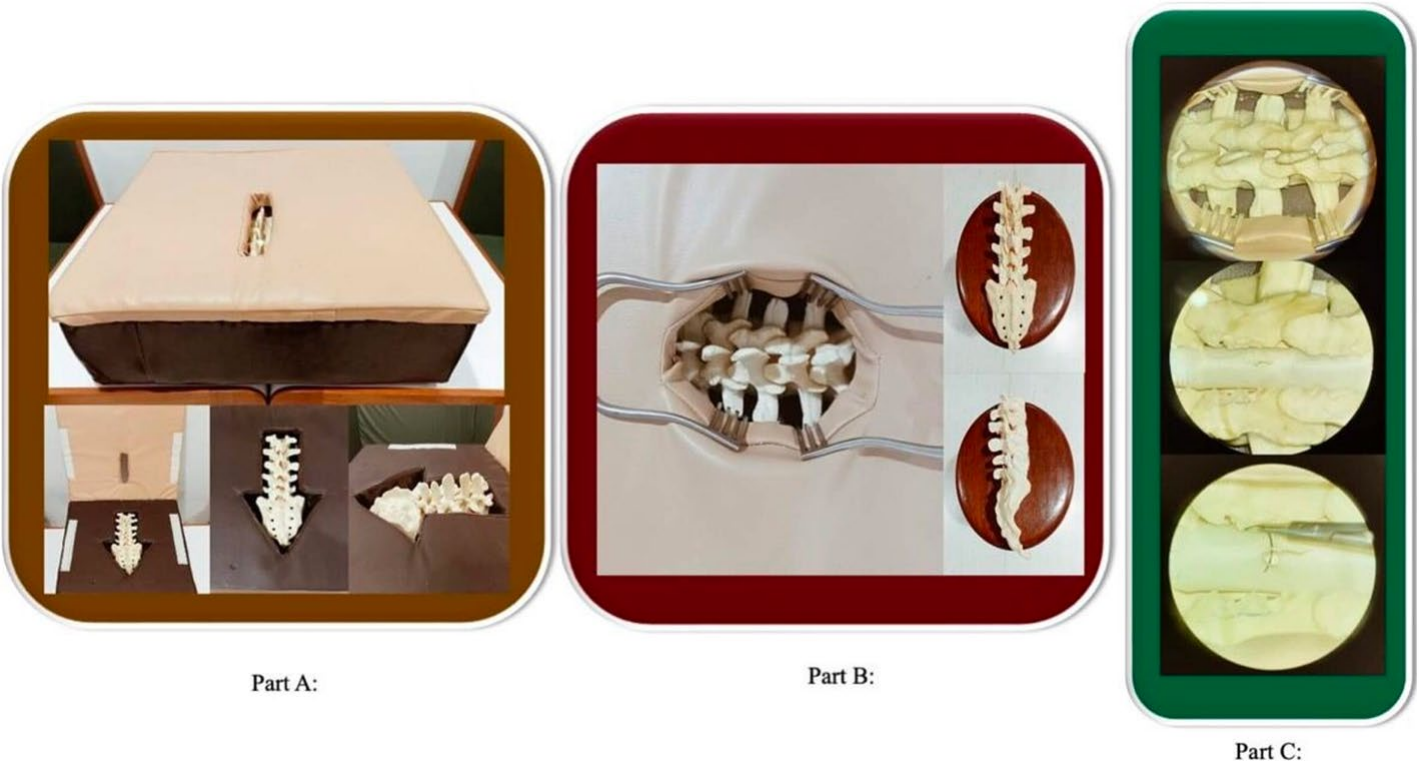
Spine surgery simulator. Part **A** shows the two components made of foam covered with rexine, which opens like a book. The lower part has space in the middle for spine model, and the upper part has a slit like opening depicting incision. Part **B** shows the widened opening by applying lumbar spine retractors for better exposure. The spine model is made of saw-bone material. Part **C** shows pictures taken under the microscope before and after laminectomy. Latex glove was used to simulate dura, and it was pasted on a 5cc plastic syringe with a window in middle. Suture is being applied using 6’0 Prolene under microscope as seen above. Each simulator had L1 to S1 spine model, and two residents worked on one model during the bootcamp.

To prevent observer bias, participants were assigned stations in such a way that the supervising faculty did not belong to their parent hospital. Each participant attempted each skill at least thrice during the course. The 1st and 3rd attempts were scored on the following domains: respect for tissue, time and motion, instrument handling, flow of operation and forward planning, knowledge of procedure, overall impression, and quality of work. Residents were asked to mention the number of times they had previously performed the skills being evaluated in the bootcamp in hospitals on their patients. An indigenously built low-cost ($60) spine surgery simulator was used in the bootcamp, which is shown in Fig. 2.

### Feedback

Detailed feedback was obtained from residents and faculty on the relevance and usefulness of the topics and skills covered in the bootcamp. This was done using a standard 5-point Likert scale (from 1-5, where 1 represented strongly disagree and 5 depicted strongly agree).

### Data analysis

Responses from the three study tools were recorded using the REDCap software for each participant. The data was only shared with the primary research team conducting the study and protected in a password encrypted file. Data was analyzed using StataCorp. 2019 (Stata Statistical Software: Release 16. College Station, TX: StataCorp LLC). Continuous data has been presented as frequencies. Statistical means, standard deviations and means have been compared using Mann-Whitney U test. Categorical data has been presented as frequencies and proportions and associations were assessed through Fisher Exact test. Values of *p* less than 0.05 were considered statistically significant.

## Results

All 22 participants of the bootcamp gave informed consent to be part of the study. The raw data of the study can be seen in supplementary file 1. Around two-third residents hailed from public sector hospitals (*n*=15; 68.18%), while 7 (31.82%) were from private institutes. Mean age of the participants was 28.42 +/- 0.31 years. There was equal gender distribution with 11 males and 11 females. Only one participant (4.55%) had previously attended a neurosurgical skills workshop. Three residents belonging to the same institute agreed that they had a neurosurgical simulation lab at their center.

**Fig 3:**
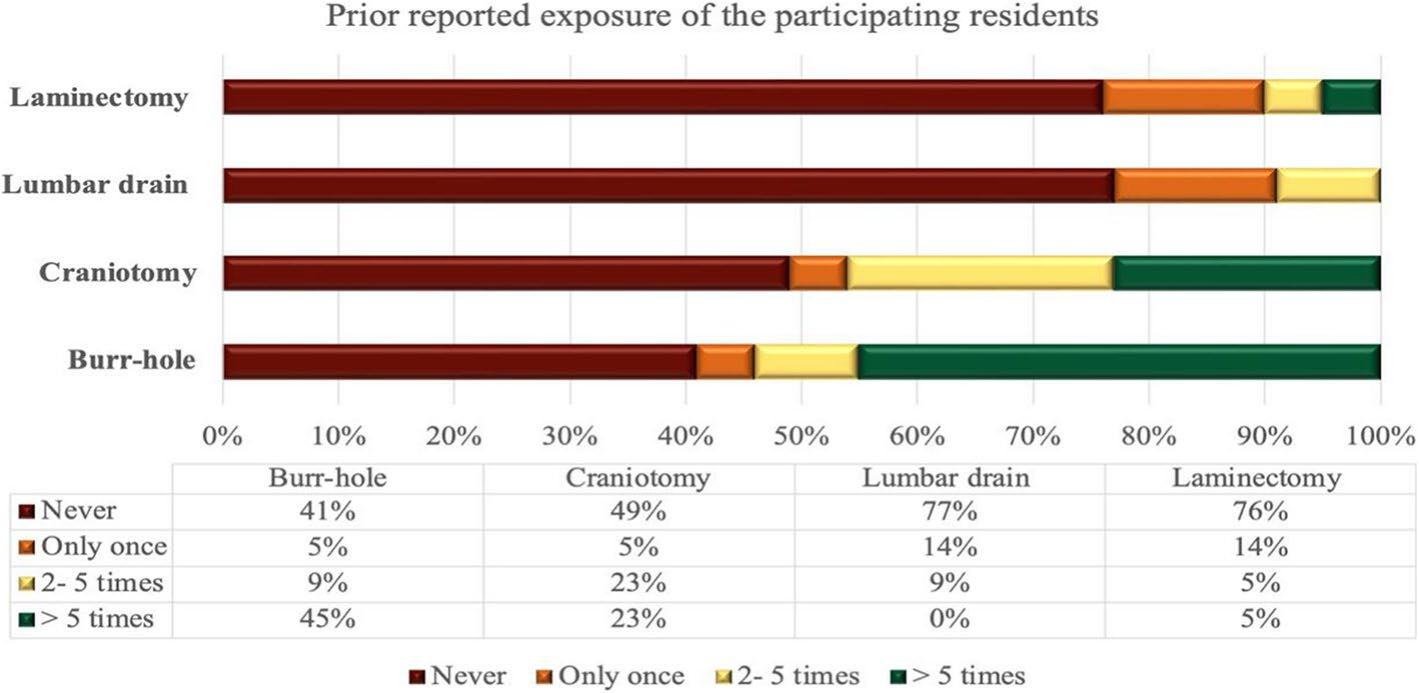
Prior experience of residents in performing the four operating skills that were evaluated

All residents performed the 12 hands-on skills; however, assessment was only done for two cranial and two spinal procedure skills. Figure 3 depicts the prior exposure of residents to the 4 practical skills that were evaluated. Table 2 shows details of prior exposure to performing the skills, and the comparison of outcomes on the 1^st^ and 3^rd^ attempts, of cranial and spinal skills.

**Table 2:** Cranial skills’ evaluation

S. No	Domains	Burr-hole 1st attempt	Burr-hole 3rd attempt	*P*-value	Cranioto-my 1st attempt	Cranioto-my 3rd attempt	*P* -value
1	Respect for tissue	3	4	< 0.001	3	4	< 0.001
2	Time and motion	3	4	< 0.001	3	4	< 0.001
3	Instrument handling	3	4	< 0.001	3	4	< 0.001
4	Knowledge of instruments	3	4	< 0.001	3	4	< 0.001
5	Use of assistants	4	4	0.017	3	4	< 0.001
6	Flow of operation and forward planning	3.5	4	< 0.001	3	4	< 0.001
7	Knowledge of specific procedure	3	4	< 0.001	3	4	< 0.001
Spinal skills’ evaluation							
	Domains	Lamin- ectomy 1st attempt	Lamin- ectomy 3rd attempt	*P*-value	Lumbar drain 1st attempt	Lumbar drain 3^rd^ attempt	*P*-value
8	Respect for tissue	2	4	< 0.001	2	4	< 0.001
9	Time and motion	2	4	< 0.001	2	4	< 0.001
10	Instrument handling	2	4	< 0.001	2	4	< 0.001
11	Knowledge of instruments	3	4	< 0.001	2	4	< 0.001
12	Use of assistants	2.5	3.5	< 0.001	2	3	< 0.001
13	Flow of operation and forward planning	2	4	< 0.001	2	4	< 0.001
14	Knowledge of specific procedure	2	4	< 0.001	2	4	< 0.001

Faculty and residents were requested to provide feedback on the two-day bootcamp at its conclusion. Twenty-one residents and 12 faculty provided feedback. An overwhelmingly positive feedback was received, with nearly all activities rated greater than 4 for both relevance and usefulness on the Likert scale. Details are shown in Table 3.

**Table 3:** Faculty and resident rating of the relevance and quality of the bootcamp

**Hands-on Training Sessions**	**Relevance**		**Usefulness**	
	**Faculty Rating**	**Resident Rating**	**Faculty Rating**	**Resident Rating**
External Ventricular Drain insertion	4.4	4.35	4.5	4.4
Intracranial pressure monitoring	4.5	4.3	4.5	4.35
Ventriculo-peritoneal shunt insertion	4.4	4.4	4.2	4.45
Neuro-navigation for cranial cases	4.5	4.25	4.3	4.25
Simulated burr-holes on 3D skulls	4.7	4.55	4.8	4.5
Simulated craniotomy on 3D skulls	4.6	4.5	4.7	4.65
Handling of the operative microscope	4.6	4.7	4.7	4.65
Microdissection of cow brain	4.3	4.7	4.3	4.7
Simulated lumbar drain insertion	4.5	4.4	4.7	4.6
Simulated Lumbar laminectomy	4.5	4.6	4.6	4.7
Simulated spine dural closure	4.3	4.35	4.3	4.4
Basic Neurosurgical instrumentation	4.6	4.15	4.5	4.3

### Cost

**Fig 4:**
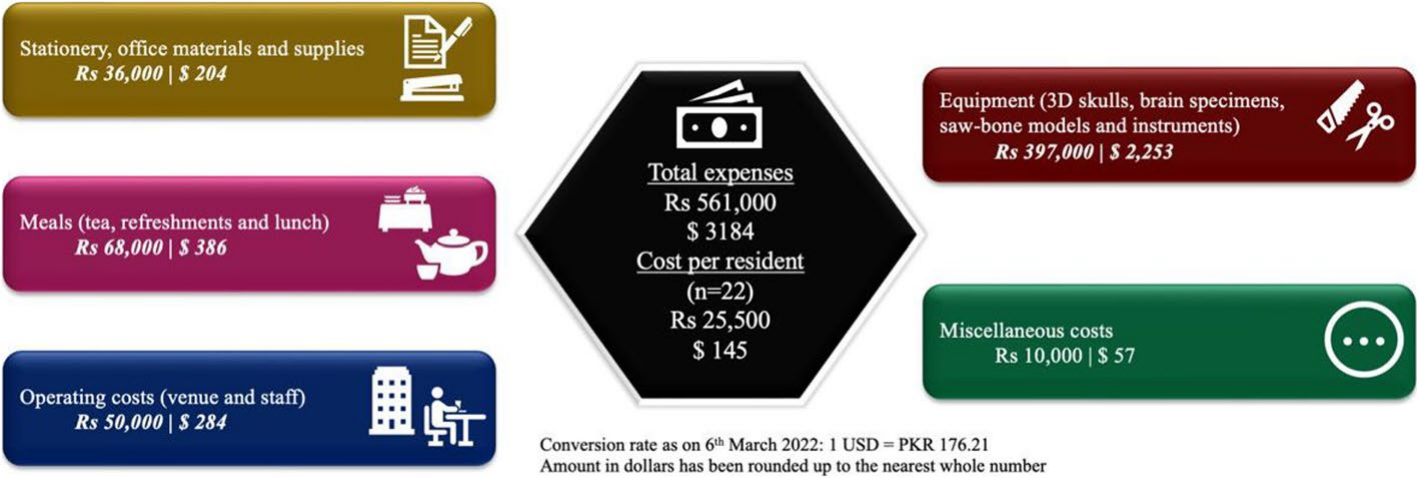
Details of bootcamp cost

The cost of one 3D skull was $57 and the spine simulator with saw-bone model cost $60. We had one set of instruments for laminectomy at each station, which cost $42/set. We reused perforators and cutters from the OR after sterilization, and saved the charges. After adding miscellaneous charges and venue/food expenses, the cost per participant was $145. Some items had been provided free of charge by the vendors, like lumbar drains, electronic craniotome and 6’0 proline sutures. Refer to Fig. 4.

## Discussion

This study is the first comprehensive report and quantitative evaluation of the inaugural neurosurgery bootcamp, conducted in the world’s 5^th^ most populous country. Our course curriculum comprised of 12 neurosurgical skill stations. We evaluated the four fundamental practical skills which nearly half of the residents performed for the first time during the course. The trainers observed significant improvement in residents’ performance under supervision for all aspects of the cranial and spinal skills being assessed. Almost all residents and faculty who gave feedback, rated the curriculum useful and relevant to their training requirements.

The last two decades have witnessed a robust change in clinical teaching methodologies. Bootcamps and simulation have now become a regular feature of junior resident training in most high-income countries. The Society of Neurological Surgeons (SNS) introduced the concept of bootcamp for first- and second-year neurosurgery residents in the United States (US) in 2009 [7]. Eighteen junior residents from five different institutes were taught fundamental neurosurgical concepts and skills in the two-day event [7]. The effectiveness of this bootcamp was gauged via online forms filled by the faculty and participants right after the bootcamp for relevance and usefulness of the curriculum [7]. A series of regional bootcamps were held throughout US in 2010 for junior residents, comprising of didactic lectures covering common topics, and hands-on training of basic neurosurgical skills [5].

Selden et al. conducted a 6-month post-bootcamp appraisal of knowledge retention and skills utility [12]. Ninety-nine percent of the 164 participants of this survey believed that the bootcamp played a significant role in improving patient care [12]. Effectiveness of their curriculum was also proved by the observation of a statistically significant knowledge retention rate in these residents. Their curriculum was later approved by ACGME and was implemented across the US. The curriculum for our bootcamp was adopted from the one published by Selden et al., with some modifications from local experts. In the post-bootcamp online feedback, an overwhelming majority of residents and faculty rated the topics and skills to be both relevant and useful.

As an expansion of the North American neurosurgery training model, neurosurgeons from the United States and Bolivia jointly organized the first neurosurgery bootcamp in South America which was attended by 24 neurosurgery residents [8]. Ament et al. published an account of their experience and the feedback received from the participants and faculty [8]. Although they incurred a high cost of $40,000, this was the first bootcamp conducted in South America and was very well received in the Bolivian neurosurgical community. In 2018, Rock et al. published their experience of organizing the first neurosurgery bootcamp for residents hailing mostly from seven middle- and high-income Southeast Asian countries, in Myanmar [9]. This was a collaborative effort between the Myanmar Neurosurgical Society, Foundation for International Education in Neurosurgery, Society for Neurological Surgeons, The University of Medicine 1 in Yangon, Myanmar, and the Henry Ford Department of Neurosurgery [9]. Their course was a modification of the original SNS curriculum incorporating advanced topics as well. The Myanmar bootcamp was a rapid neurosurgical skills’ review course, where 40 residents with variable neurosurgical training periods participated. In 2020, Lepard et al. reported a comprehensive perspective on the Myanmar bootcamp experience from 45 residents [14]. Their results highlighted the unmet need of neurosurgical care, and the requirement of sustainable training initiatives to overcome the issue of reduced neurosurgical workforce. They proposed replication of the collaborative effort between high-income and under-privileged countries as a workable solution [14].

Being an effective educational strategy in improving learners’ skills, knowledge and confidence, bootcamps have been widely adopted in neurosurgery training in all high-income countries. Some major hinderances in replicating this model in LMIC are costs, unavailability of cadavers and the required equipment and significant variability in training standards at different centers. Our study reports a sustainable low-cost simulation-based solution which can be easily reproduced in a low-resource setting. Since the surgical instruments can be reused after an initial expenditure, subsequent bootcamps will incur even lower expenditure. The indigenously designed spine surgery simulator can be modified for variable difficulty level by increasing depth. Although we did not evaluate the simulated dural closure in the bootcamp, as it required more 3 attempts to be sufficiently able, the model can be used by residents for practice in lab.

This was all the participant’s first experience of attending a bootcamp. Although we tried to limit the observer bias in skills’ assessment by pairing residents with faculty from different institutes, we can expect some partiality in evaluations, because the same faculty taught and later assessed the residents. Since we had a small sample size, we could not gauge the impact of any prior surgical exposure on the residents’ performance in the bootcamp. The residents and faculty reported favorable experience of using the indigenously designed spine surgery simulator, but it has not yet been systematically validated for formal training. Furthermore, the non-uniform curriculum and highly variable surgical exposure in different centers in our country could have impacted residents’ performance during the bootcamp.

## Conclusion

Through this study, we ascertained the applicability and efficacy of simulation-based learning, to further refine and tailor future educational activities/curriculum for neurosurgery trainees. We have proposed a low-cost sustainable training model for LMIC. This will also lay the groundwork for a future national curriculum that focuses on developing core competencies required from a neurosurgery resident, irrespective of their training center.

## Supplementary Information

**Additional file 1.**

## Data Availability

The datasets generated and/or analyzed during the current study have been included in the results section and are publicly available. A raw data file has been added in the supplementary material and is publicly accessible.

## Abbreviations

ABS: Acrylonitrile Butadiene Styrene
ACGME: Accreditation Council for Graduate Medical Education
AKUH: Aga Khan University Hospital
CPSP: College of Physicians and Surgeons Pakistan
GDP: Gross Domestic Product
LMIC: Low and middle-income countries
mOSATS: modified Objective Structured Assessment of Technical Skills
PG-Y1: Post-graduate year-1
SNS: Society of Neurological Surgeons
